# Early diagnostic accuracy of immature platelet fraction for sepsis: a systematic review and meta-analysis

**DOI:** 10.1016/j.htct.2026.106476

**Published:** 2026-05-30

**Authors:** Sinta Wiranata, Ida Bagus Amertha Putra Manuaba, I Gede Putu Supadmanaba, I Putu Yuda Prabawa, Anak Agung Wiradewi Lestari

**Affiliations:** a Department of Clinical Pathology, Faculty of Medicine, Universitas Udayana, Prof. Dr. I.G.N.G. Ngoerah Center General Hospital, Bali, Indonesia; b Medical and Health Education, Faculty of Medicine, Universitas Udayana, Denpasar, Bali, Indonesia; c Biochemistry Department Faculty of Medicine, Universitas Udayana, Denpasar, Bali, Indonesia; d Department of Clinical Pathology, Faculty of Medicine, Universitas Udayana, Udayana University Hospital, Bali, Indonesia

**Keywords:** Early detection, Immature platelet fraction, Sepsis

## Abstract

**Background:**

The immature platelet fraction, a rapid and accessible indicator of bone marrow thrombopoietic activity, is considered a possible biomarker for the early diagnosis of sepsis. This study aims to evaluate the diagnostic precision of immature platelet fraction testing for the early identification of sepsis. This is, to our knowledge, the first systematic review and meta-analysis addressing this problem.

**Methods:**

A literature search was carried out on the PubMed and Scopus databases from August 1 to August 8, 2025. Studies that met the inclusion criteria were analyzed to calculate sensitivity, specificity, diagnostic odds ratio, Deeks’ funnel plot, and summary receiver operating characteristic. Sub-group analysis was carried out to evaluate differences in immature platelet fraction diagnostic performance in adults excluding pediatrics data.

**Results:**

Three studies published between 2013 and 2021 satisfied the selection criteria and were incorporated into the analysis. Meta-analysis indicates that the immature platelet fraction possesses moderate diagnostic accuracy for identifying sepsis. The pooled sensitivity was 74% (95% CI: 53%–87%), and the pooled specificity was 70% (95% CI: 48%–85%). The combined diagnostic odds ratio was 6.54 (95% CI: 2.90–14.76). Moderate heterogeneity was observed between the included studies (I^2^ = 59.57%; τ^2^ = 0.30).

**Conclusions:**

Immature platelet fraction may represent a complementary biomarker for sepsis, but limited evidence and methodological heterogeneity necessitate cautious interpretation as these pooled estimates were derived from a limited number of studies. Further standardized prospective studies are required to establish its clinical utility.

## Introduction

Sepsis is a serious worldwide health concern, especially in developing countries. Sepsis, by definition, is severe and fatal organ failure caused by the body's reaction to an infection that it cannot control [1]. Globally, sepsis accounts for an estimated 48.9 million cases and 11 million deaths annually, representing 19.7% of all fatalities worldwide. Furthermore, data analysis across 109 million individual death records underscores the substantial global burden of the condition [2]. Purba et al. reported a case fatality rate among patients receiving treatment in intensive care units of 69% [3]. The mortality rate for severe sepsis and septic shock can range from 25% to 70%, making early identification imperative [4]. The elevated death rate underscores the importance of early identification to promptly and correctly commence therapy. Given the central role of platelet activation and consumption in sepsis-induced coagulopathy, biomarkers reflecting real-time thrombopoietic activity may provide additional diagnostic value. The immature platelet fraction (IPF), which reflects increased bone marrow platelet release in response to peripheral consumption, represents a readily available hematological parameter that might complement existing sepsis biomarkers [5]. However, the diagnostic performance of the IPF for early sepsis identification has not been systematically evaluated.

Timely identification of sepsis necessitates an integration of clinical assessment and laboratory indicators. Frequently employed measures encompass procalcitonin (PCT), C-reactive protein (CRP), lactate, and a complete blood count [6,7]. PCT serves as a measure for systemic bacterial infection, CRP signifies an acute inflammatory response, and lactate reflects tissue hypoperfusion. Nevertheless, the use of these parameters has constraints: PCT and CRP lack specificity for coagulation activity. The platelet count alone indicates the quantity of platelets and does not represent the activity of bone marrow production. All these indicators remain of low efficacy in preventing multiorgan dysfunction syndrome resulting from sepsis. This syndrome is initiated by persistent microcirculatory abnormalities, which ultimately lead to death [8, 9, 10].

Among the numerous mechanisms contributing to microcirculatory disorders, dysfunctions in the coagulation system are pivotal in sepsis-induced organ damage [11]. Despite the contentious nature of microthrombi in sepsis, microvascular alterations may transpire even in the absence of evident thrombotic occurrences [12]. The severity of a patient's condition may deteriorate when systemic inflammation triggers coagulation factors and platelets [13]. This highlights the necessity of including coagulation indicators within the laboratory assessment for sepsis diagnosis [5].

Sepsis triggers Tumor Necrosis Factor-alpha (TNF-α), interleukin(IL)-1β, and IL-6 as inflammatory mediators, which activate the vascular endothelium [14]. The activation of endothelial cells leads to the release of tissue factors that trigger the coagulation cascade, resulting in the formation of microthrombi and the consumption of significant quantities of platelets, frequently culminating in disseminated intravascular coagulation. The reduction in platelet count prompts the bone marrow to enhance thrombopoiesis and release an IPF into the bloodstream, resulting in an elevated intravascular platelet count [14,15]. A high IPF value in sepsis signifies that thrombocytopenia results from platelet consumption or destruction, with bone marrow function remaining intact. In contrast, a low IPF shows a failure in bone marrow production.

The IPF is the percentage of reticulated platelets that still contain residual RNA from megakaryocyte formation and is a direct indicator of thrombopoietic activity in the bone marrow [16,17]. IPF testing may be conducted automatically with fluorescence flow cytometry alongside a complete blood count. The benefit of the IPF compared to other metrics is in its capacity to identify alterations in platelet production at an early stage, prior to a substantial decline in total platelet count, hence serving as a biomarker for the early detection of thrombocytopenia in sepsis [18].

Numerous studies indicate that the IPF has great diagnostic accuracy for the early identification of sepsis [19, 20, 21, 22, 23, 24, 25, 26, 27]. The results for diagnostic testing are still pending for some individuals; therefore, this study seeks to ascertain the definitive outcomes. However, existing evidence includes both adult and pediatric populations, which may influence diagnostic performance due to age-related hematological differences. This study sought to evaluate the diagnostic precision of IPF testing for the early identification of sepsis. This is, to our knowledge, the first systematic review and meta-analysis addressing this problem.

## Methods

### Search strategy

This study followed the PRISMA (Preferred Reporting Items for Systematic Reviews and Meta-Analyses) guidelines for reporting on diagnostic test accuracy of studies [28]. PubMed and Scopus databases were searched for relevant articles using keyword searches tailored to the topic of the IPF in sepsis. PubMed and Scopus were selected due to their broad coverage of biomedical, laboratory medicine, and diagnostic accuracy literature. These databases are widely used in systematic reviews of diagnostic test accuracy studies and were considered sufficient to capture relevant evidence on IPF in sepsis. The search period, from August 1 to August 8, 2025, used keywords such as Reticulated Platelet OR Immature Platelets OR Immature Platelet Fraction OR IPF AND sepsis. No restrictions were applied regarding publication year or article type. All references found were entered into the Mendeley reference manager software, with duplicate records being removed.

### Study selection

The research selection procedure was separately executed by two authors (S.W., I.P.Y.P.) employing a blinded review methodology. Disputes were settled through discussion until a consensus was achieved, augmented by the perspective of a third author (A.A.W.L.). The initial round of selection comprised the examination of titles and abstracts, followed by a comprehensive assessment of papers that fulfilled the preliminary criteria. This manuscript was prospectively registered in the PROSPERO database under the registration ID number CRD420251123676.

**Figure 1:**
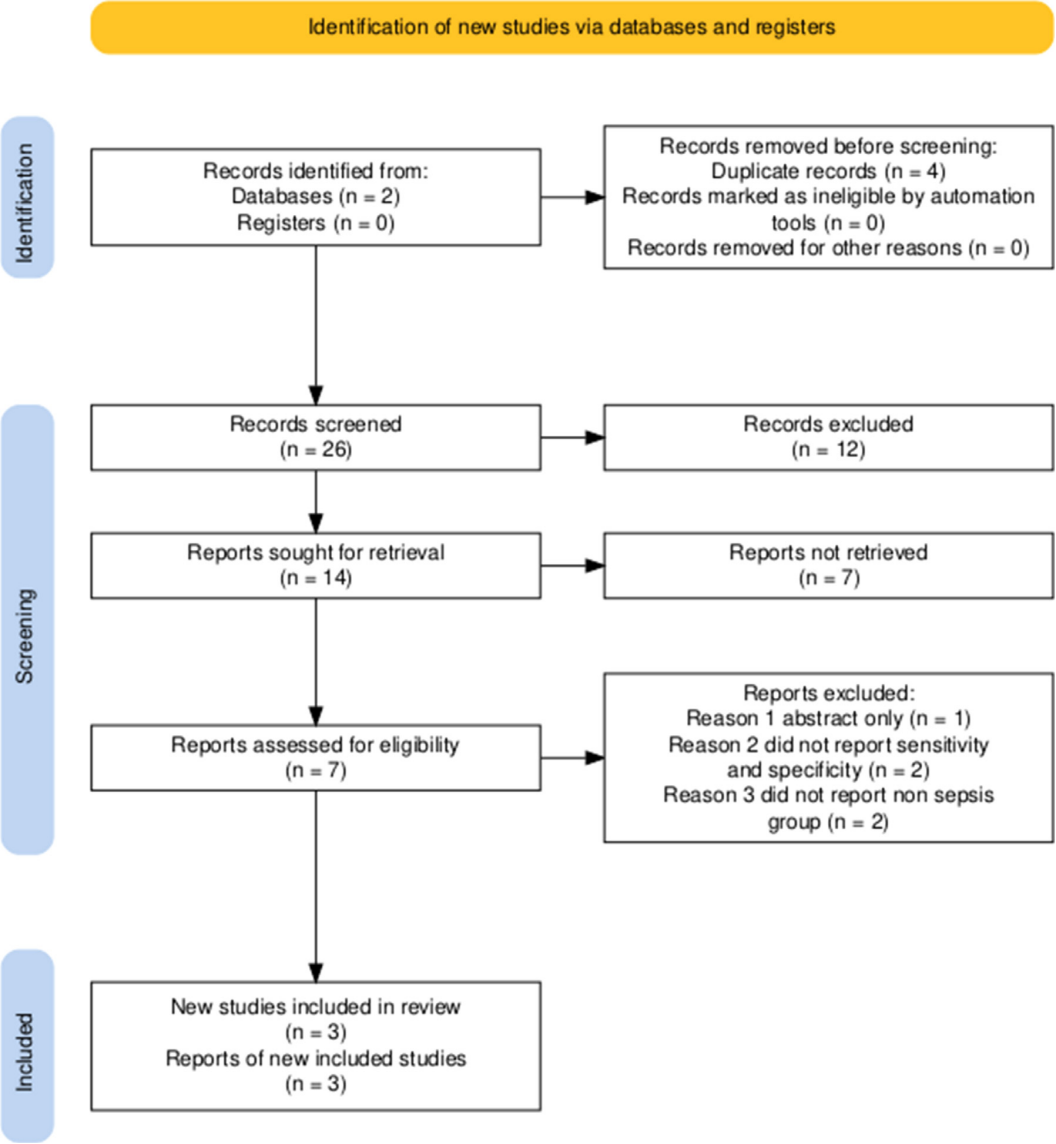
Flow diagram of the literature search and selection process.

Included studies were original investigations documenting IPF examination results in patients who exhibited no sepsis criteria at arrival but developed sepsis during the research period of the original article. The exclusion criteria encompassed: review articles, letters to the editor, commentaries, conference abstracts, case reports, small case series (fewer than 50 cases), articles in languages other than English, *in vitro* or animal studies, studies pertaining to sites/sample types unrelated to sepsis, studies with overlapping data from larger publications, and studies focused on non-sepsis diseases. Several potentially relevant studies could not be included due to inaccessible full texts and insufficient diagnostic data, which may have limited the completeness of available evidence (Figure 1).

### Data extraction

Data extraction was performed independently by two authors (S.W., I.P.Y.P.), with discrepancies resolved through consensus of three authors (S.W., I.P.Y.P., A.A.W.L.). Extracted data included: first author's name and year of publication, country, study period, study design, patient age, total number of sepsis cases and controls, sensitivity, specificity, the IPF cut-off, and the number of positive and negative cases in both the sepsis and control groups.

If one of the data points was unavailable, the sensitivity and specificity calculations were carried out using the diagnostic test calculator in Review Manager version 5.4.

### Study quality assessment

Study quality was evaluated using the QUADAS-2 (Quality Assessment of Diagnostic Accuracy Studies 2) instrument in four domains: study selection, index test, reference test, and sampling flow and timing.

### Statistical analysis

Statistical analyses were performed using Stata software version 17. For each study, sensitivity and specificity were calculated univariately using random effects. The combined diagnostic odds ratio (DOR) was calculated using a random effects model of the IPF test in diagnosing sepsis. A summary receiver‐operating characteristic (SROC) curve was generated by plotting individual and pooled sensitivity and specificity points to assess overall diagnostic accuracy. Subgroup analyses were performed based on the adult sepsis population. Finally, potential publication bias was evaluated using a Deeks’ funnel plot, recommended for systematic reviews of diagnostic test accuracy studies.

## Results

### Literature search: study characteristics

The study identification and selection process for this systematic review began with a literature search of two databases (*n* = 2) without additional reference to register sources. Of the 30 records found, four were removed due to duplication. No records were removed due to automatic ineligibility or other reasons. The remaining 26 records were then screened based on the title and abstract, yielding 14 potentially relevant reports. Of these, seven reports were not accessible for full-text review. A total of seven reports were fully assessed for eligibility. A further four were eliminated for the following reasons: one report was available only in abstract form, two reports did not report sensitivity and specificity values, and two reports did not include a non-sepsis comparison group.

Finally, three studies from Italy, South Korea, and Turkey, published between 2013 and 2021, met the inclusion criteria for this systematic review and meta-analysis [19, 20, 21]. The IPF cutoff points varied across the studies. The total sample size included in this review was 451 patients. Additional methodological details regarding the characteristics of each study, reference standard for sepsis diagnosis, and the timing of the IPF measurement are summarized in Table 1. All included studies measured the IPF at or near hospital admission and all analyzed studies used IPF testing to detect sepsis in a population of patients with suspected infection or critical illness.

**Table 1:** Study characteristics of the included analysis.

Study	Country	Study design		Sepsis Definition	Timing IPF	ROC compare content	Cutoff values of IPF%	No. of patients		Age (mean ± SD/median)	
								Case	Control	Case	Control
De Blasi et al. 2013^19^	Italy	Prospective		Sepsis-2	1 hour after ICU admission	Sepsis versus non-sepsis	4.7	33	31	59.6 (19.3)	55.4 (15.9)
Park et al. 2016^20^	South Korea	Cross‐sectional		Sepsis-2	At the time of admission	Sepsis versus non-sepsis	3.1	215	47	66 (20–92)	52 (20–84)
Turkmen et al. 2021^21^	Turkey	Prospective		Sepsis-2	At the time of admission	Sepsis versus non-sepsis	2.7	78	47	21 (2–86)	23 (2–120)

IPF: Immature platelet fraction; ROC; Receiver operating characteristic; SD: Standard deviation; ICU: Intensive care unit.

### Quality assessment and bias analysis

**Figure 2:**
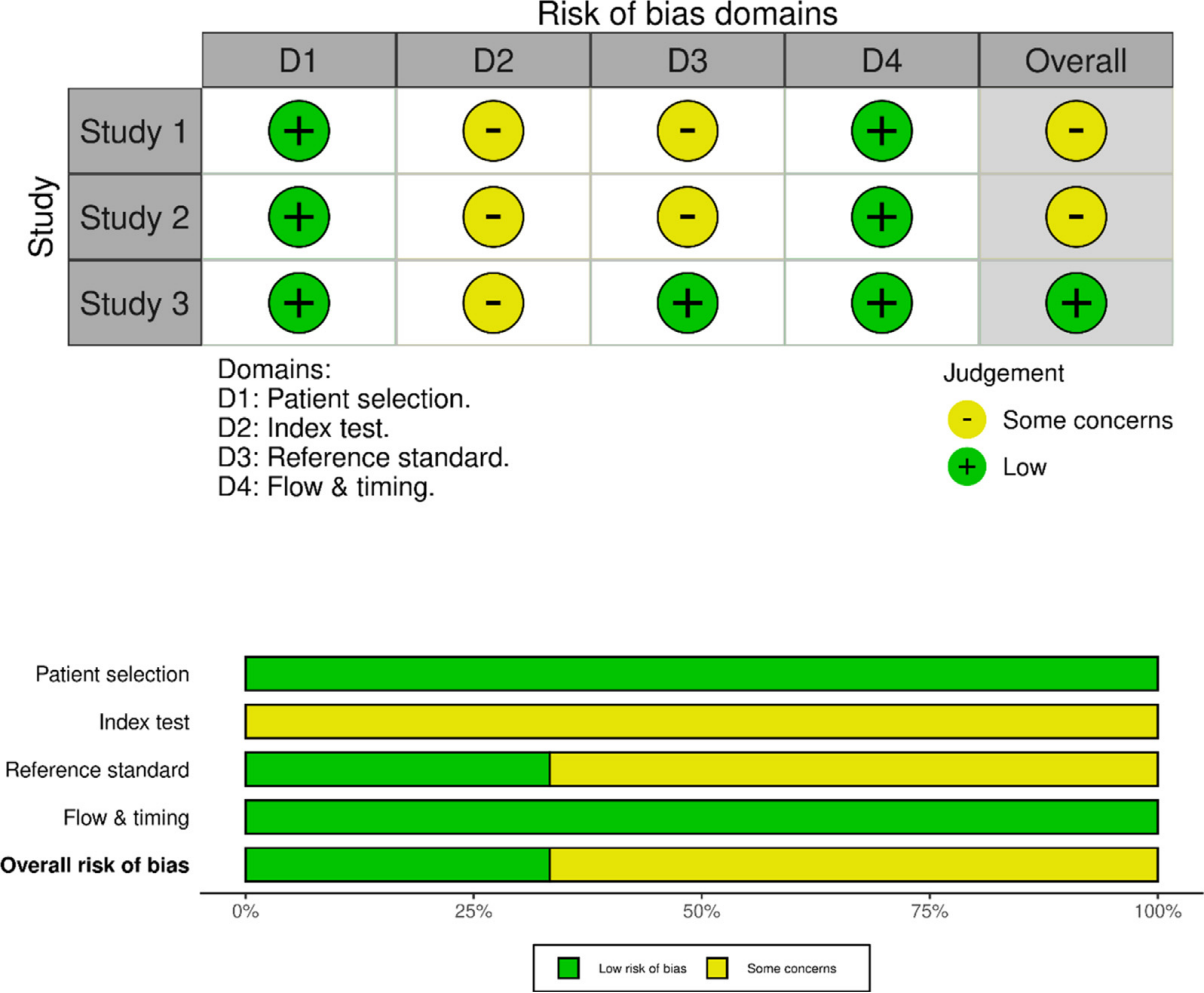
Study quality assessment using the Quality Assessment of Diagnostic Accuracy Studies 2 (QUADAS-2) tool.

Based on the QUADAS-2 assessment [29], two studies were assessed as having an ‘unclear risk of bias’ in the Index Test and Reference Standard domains due to insufficient information regarding blinding procedures and details of the reference test implementation. Overall, the methodological quality of all three studies was considered of low concern (Figure 2).

### Diagnostic accuracy

**Figure 3:**
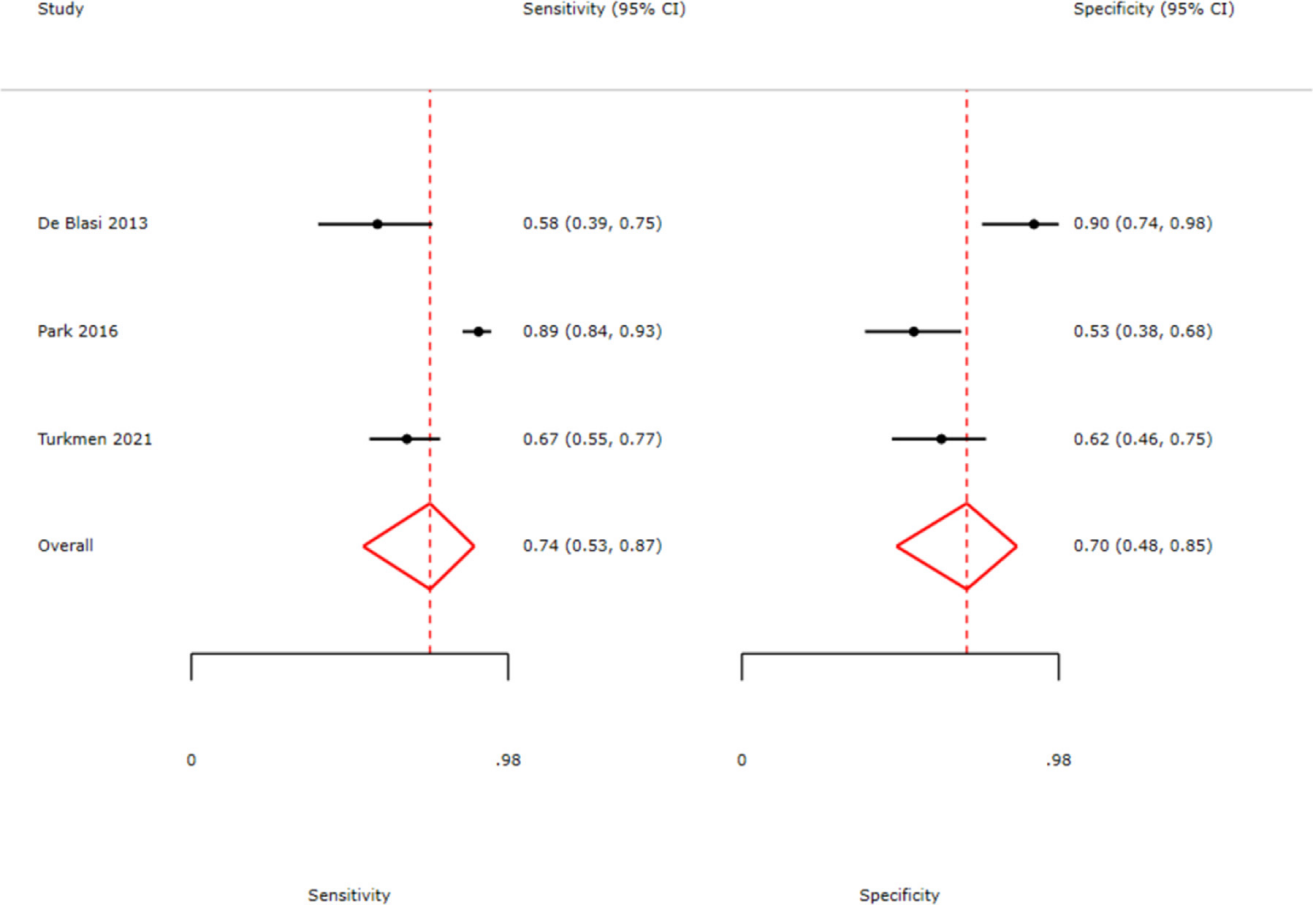
Forest plots of pooled sensitivity and specificity for sepsis diagnosis. The analysis illustrates the diagnostic performance of the immature platelet fraction across three included studies. Point estimates (black circles) and 95% confidence intervals (horizontal lines) are provided for each study. The red diamonds represent the pooled summary estimates, yielding an overall sensitivity of 0.74 (95% CI: 0.53–0.87) and a specificity of 0.70 (95% CI: 0.48–0.85).

Data from eligible studies were analyzed to calculate the diagnostic accuracy of the IPF test for sepsis. A forest plot of sensitivity and specificity is shown in Figure 3. The pooled sensitivity of the IPF test for diagnosing sepsis was 74% (95% Confidence interval [95% CI]: 53%–87%), and the pooled specificity was 70% (95% CI: 48%–85%).

**Figure 4:**
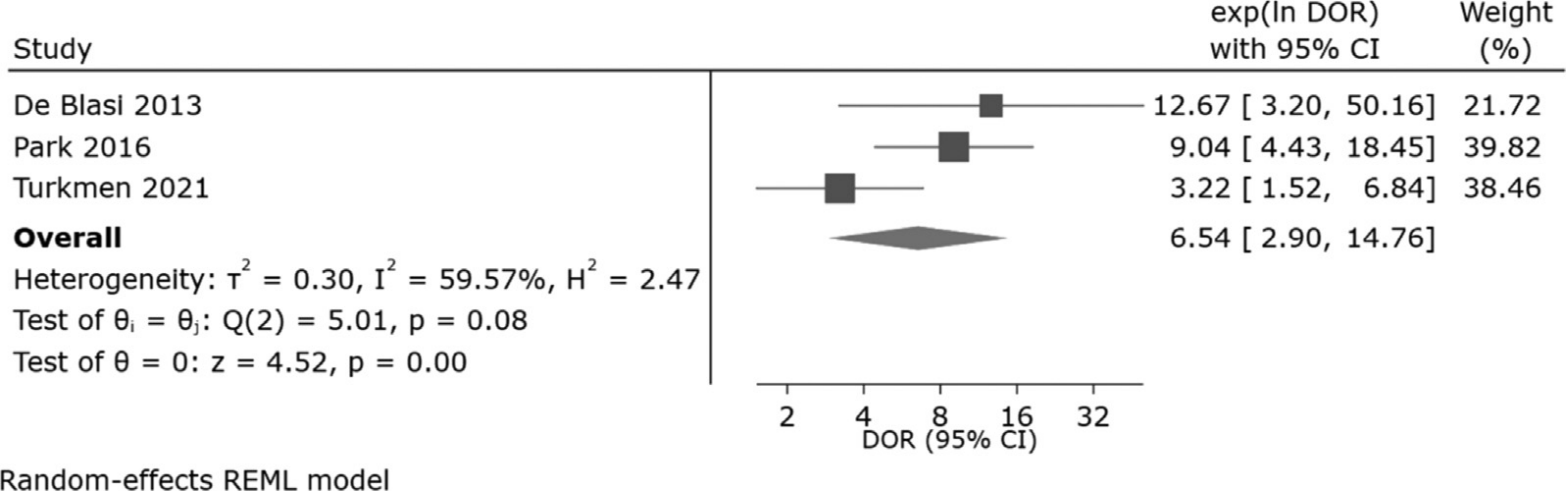
Forest plot of the diagnostic odds ratio (DOR) for immature platelet fraction (IPF) in Sepsis Diagnosis. Analysis, utilizing a random-effects REML model, yielded a pooled DOR of 6.54 (95% CI: 2.90–14.76). This indicates that the odds of a positive IPF result are 6.54 times higher in patients with sepsis compared to those without the condition. Moderate heterogeneity was observed (I^2^ = 59.57%; Q = 5.01; p-value = 0.08). The test of overall effect was statistically significant (*z* = 4.52; p-value < 0.001), supporting the diagnostic utility of IPF for sepsis identification.

**Figure 5:**
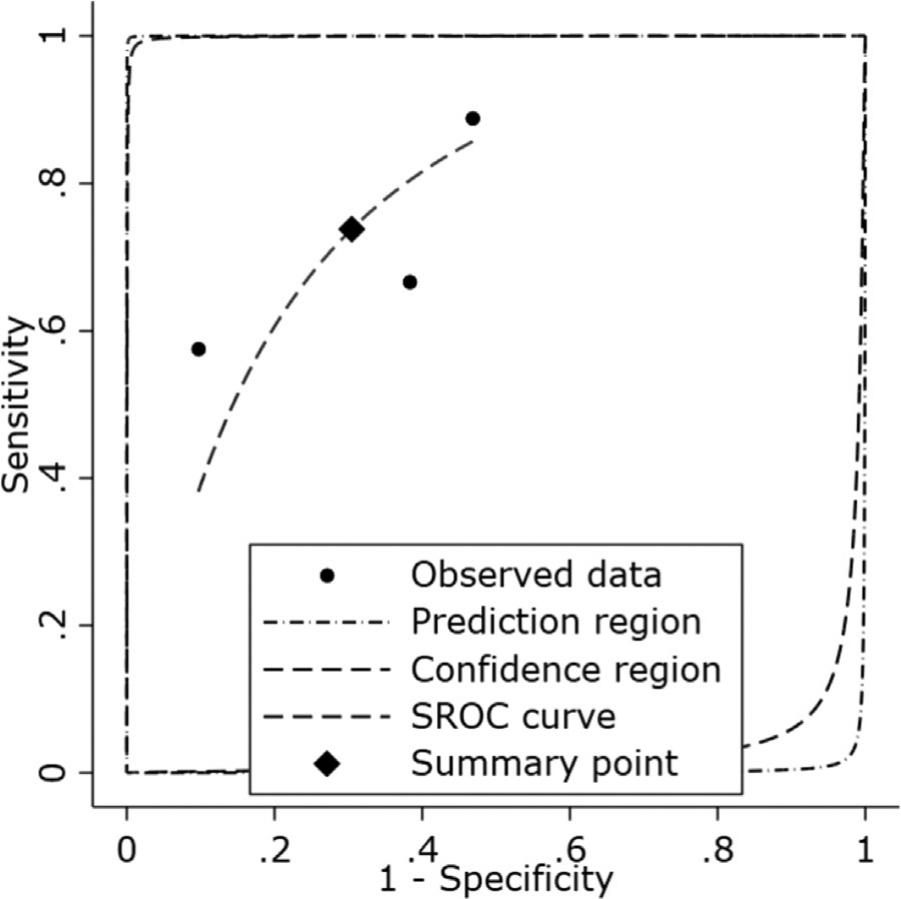
Summary receiver operating characteristic (SROC) curve of included studies. The SROC analysis integrates three studies evaluating the diagnostic accuracy of the immature platelet fraction for sepsis detection. Individual circles represent the observed sensitivity and specificity for each included study. The solid diamond indicates the pooled summary point, reflecting a combined sensitivity estimate of approximately 0.74 and a specificity of 0.70.

**Figure 6:**
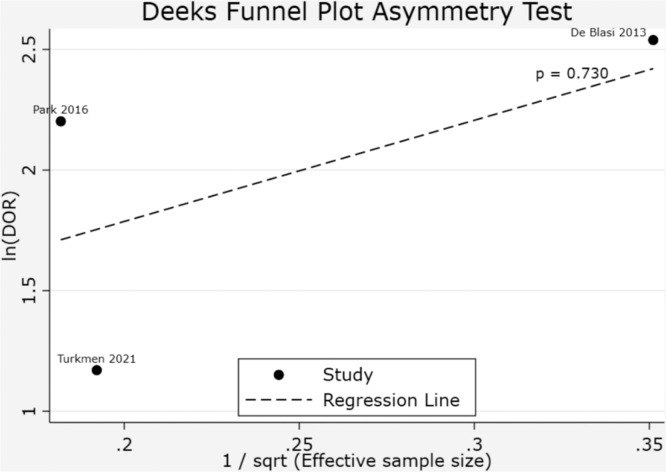
Deeks’ funnel plot assessing the potential publication bias of the three studies. No evidence for publication bias was found (*p* = 0.73).

A meta-analysis of the three studies assessing the diagnostic accuracy of the IPF for detecting sepsis yielded a pooled DOR of 6.54 (95% CI: 2.90–14.76), indicating that patients with sepsis were approximately 6.5 times more likely to have a positive IPF result than patients without sepsis (Figure 4). The combined effect significance test (Test of θ = 0) showed statistically significant results (*z* = 4.52; p-value ≤0.001), confirming that the IPF has significant diagnostic ability in differentiating patients with and without sepsis. Heterogeneity analysis (Test of θi​ = θj​) yielded a Q statistic of 5.01 (degrees of freedom = 2) with p-value = 0.08 and an I^2^ value of 59.57%. This shows that the variation in results between studies can largely be explained by differences in methodology or population characteristics, but is not large enough to obscure the combined effect results. The SROC curve shows summary points for sensitivity and specificity that fall within a reasonably broad area of the curve, supporting the ability of the IPF as a diagnostic parameter for sepsis (Figure 5). The publication bias evaluation using Deeks’ funnel plot showed a p-value = 0.730 showing there was no evidence of publication bias in the included studies (Figure 6).

**Figure 7:**
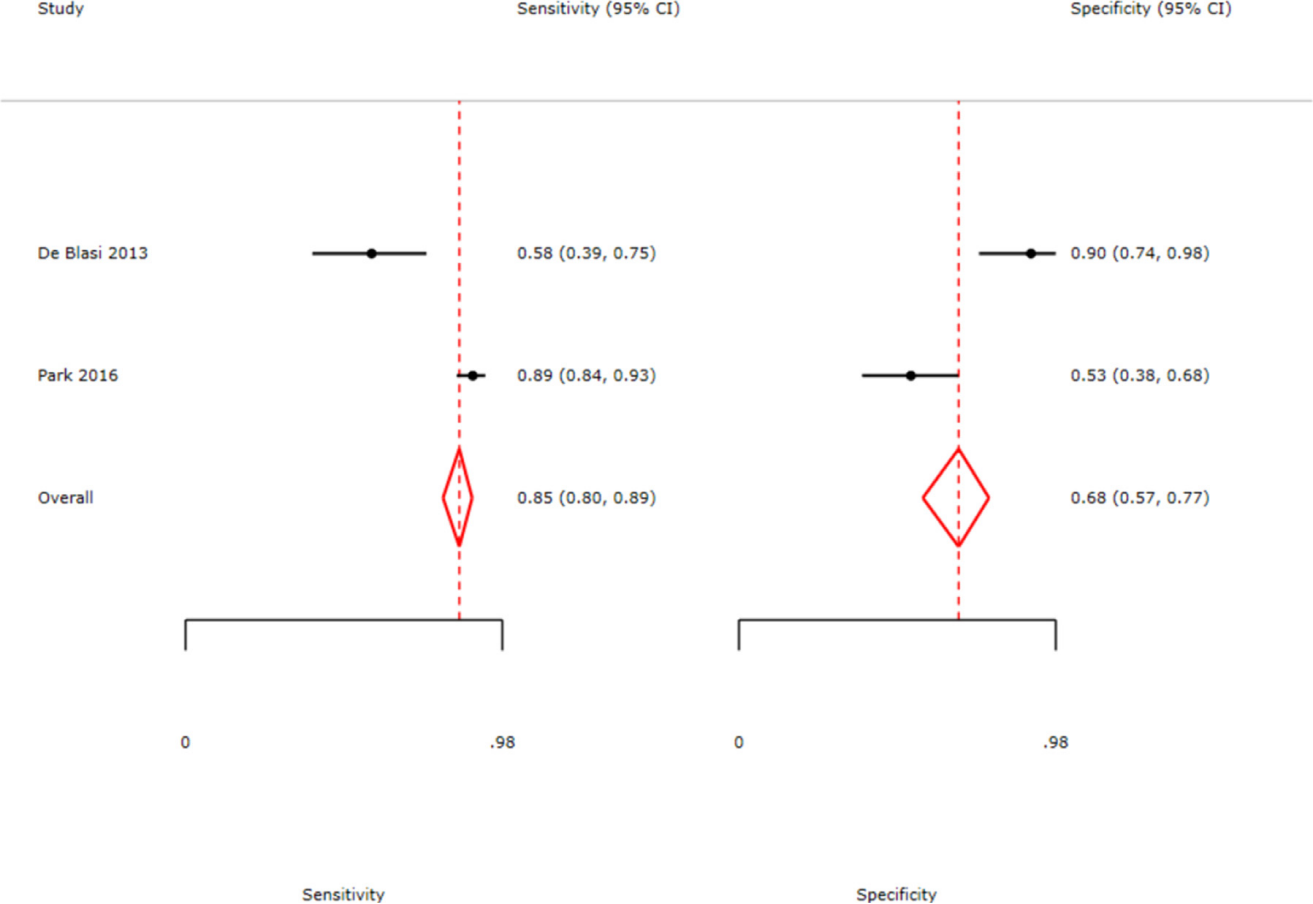
Subgroup analysis of adult sepsis patients only.

Subgroup analysis was performed to evaluate the diagnostic accuracy of the IPF specifically in the adult patient population. This was done as one of the included studies used a pediatric population, which has physiologically different hematologic characteristics than the adult population, potentially affecting the pooled estimates. [21]. Results from subgroup analyses of two studies involving adult patients showed a pooled sensitivity of 85% (95% CI: 80%–89%) and a pooled specificity of 68% (95% CI: 57%–77%) (Figure 7) [19,20]. For this evaluation, the DOR, SROC, and Deeks' funnel plot analysis were not included due to the limitations of the study. Although the sensitivity values were higher compared to the overall analysis, the specificity values were relatively similar, indicating that the IPF still exhibits moderate discriminatory ability in the adult population.

## Discussion

This meta-analysis demonstrates that the IPF holds considerable promise as a complementary biomarker for sepsis detection in mixed adult and pediatric populations. The IPF exhibited a sensitivity of 74% and a specificity of 70%. These results indicate a moderate diagnostic capacity for both identifying the condition and ruling out health in suspected cases. Clinically, this indicates that the IPF is dependable as an initial screening instrument, although not strong enough to serve as the sole test for diagnostic confirmation. These findings offer the first indication that the IPF might serve as a pertinent supplementary test, particularly when utilized with other known metrics such as PCT and CRP levels.

These findings align with literature reporting that the IPF is increased in sepsis patients due to stimulation of megakaryocytes in the bone marrow in response to platelet destruction and consumption during systemic inflammation [16]. The activation of the inflammatory response, mainly via the production of pro-inflammatory cytokines like IL-6 and TNF-α, stimulates expedited platelet maturation, leading to a notable rise in the quantity of immature platelets discharged into the bloodstream [14,15]. This situation indicates the body's compensatory efforts to maintain hemostasis while simultaneously signaling an ongoing severe inflammatory process.

In the adult population subgroup analysis, without the research on pediatric patients, sensitivity rose to 85% and specificity to 68%. This heightened sensitivity indicates that the IPF may be more effective in identifying sepsis in adult patients than in the pediatric group. The pathophysiological explanation lies in the stability of the adult hematopoietic system, which regularly reveals an increase in immature platelet formation in response to sepsis [30]. In pediatrics, physiological variability in platelet production, the development of the immune system, and varying hematopoietic responses might affect the IPF levels even without infection, hence diminishing diagnostic accuracy [30].

This finding aligns with previous reports that other inflammatory biomarkers, such as PCT and CRP, also show differences in diagnostic performance across age groups [31]. Physiological PCT levels exhibit variations across age groups, with healthy pediatric individuals presenting higher levels than healthy adults, although healthy newborns have the lowest levels. The variations in baseline levels may affect the interpretation of PCT values in sepsis diagnosis, indicating that the appropriate cut-off may differ between age groups [31]. Likewise, CRP levels may be increased owing to non-infectious reasons in young patients, including vaccinations or minor inflammatory conditions. CRP levels may rise substantially post-vaccination, with up to 85% of preterm newborns exhibiting an increase, and adults experiencing an average elevation of around 30% following influenza vaccination. This indicates that an elevation in CRP does not invariably signify infection, particularly during the post-immunization phase [32,33]. This meta-analysis aligns with many studies indicating that the IPF was considerably elevated in septic patients exhibiting neutrophilia compared to non-septic controls [34,35]. The IPF could distinguish thrombocytopenia resulting from peripheral damage (e.g., in sepsis) from thrombocytopenia caused by bone marrow hypoproduction [36, 37, 38, 39].

Sepsis triggers massive immune system activation accompanied by activation of the coagulation system [40]. A crucial mechanism is the production of microthrombi resulting from disseminated intravascular coagulation or subclinical coagulation events. This disease markedly elevates platelet consumption. Consequently, the bone marrow enhances thrombopoiesis, characterized by the discharge of immature platelets (platelet reticulocytes) into the bloodstream [40,41]. The IPF, measured by fluorescent flow cytometry on modern hematology analyzers, reflects the proportion of immature platelets in the blood and is a direct indicator of thrombopoietic activity [42].

From a clinical standpoint, the IPF offers numerous advantages: it is readily accessible and can be concurrently measured with a complete blood count on most contemporary hematology instruments; it is expeditious, yielding results within minutes, thereby facilitating prompt clinical decision-making in emergency departments or intensive care units; and it is non-invasive, negating the necessity for additional blood sampling [37]. Consequently, the data of the present study suggest that the IPF should be included in a sepsis diagnostic panel rather than utilized as an independent test. Integrating the IPF with PCT, CRP, or a clinical score like the quick Sequential Organ Failure Assessment (qSOFA) could enhance diagnostic precision and assist in patient risk stratification [23,43]. The interpretation of the IPF necessitates the evaluation of confounding circumstances, including acute bleeding, postoperative states, significant trauma, autoimmune diseases, and hematologic illnesses such as idiopathic thrombocytopenic purpura (ITP), all of which may elevate the IPF in the absence of sepsis [44]. Conversely, decreased bone marrow production resulting from chemotherapy, certain viral infections, or dietary deficits might lower the IPF count. In pediatrics, characteristics such as hematopoietic maturation, comorbidities, and nutritional conditions may further influence this value [45,46].

Several limitations must be acknowledged. The number of papers fulfilling the inclusion criteria was small (*n* = 3), resulting in a low strength of evidence. Secondly, discrepancies in research design, sepsis definitions, IPF measurement techniques, and applied cutoffs may affect the heterogeneity of the findings. Third, two of the three studies were observational, and subgroup analyses were constrained since just one study investigated a pediatric population, rendering adult-pediatric comparisons exploratory.

This meta-analysis should be interpreted with caution as the small number of included studies may reduce the precision of pooled estimates and limit generalizability. Additionally, heterogeneity may arise from differences in sepsis definitions, patient age groups, hematology analyzers, IPF measurement techniques, and applied cut-off values. Consequently, the data of this study suggest that the IPF should be included in a sepsis diagnostic panel rather than utilized as an independent test. This complementary role is consistent with the intended use of the IPF as an adjunct to established biomarkers and clinical assessment rather than a replacement. Further research should highlight the need for future standardization of IPF measurement, including harmonization of analytical platforms, calibration procedures, and clinically validated cut-off values. Establishing standardized protocols is essential to facilitate broader clinical implementation and improve the reliability of the IPF as a diagnostic adjunct in sepsis. Research should encompass a fair representation of pediatric and adult populations to elucidate variations in diagnostic performance attributable to age. Assessing the interplay of the IPF with additional biomarkers in multivariate predictive models may enhance its application in comprehensive diagnostic algorithms.

## Conclusions

The IPF may serve as a complementary diagnostic biomarker for sepsis due to its rapid availability and integration into routine hematological testing. However, the limited number of studies and methodological heterogeneity warrant cautious interpretation. Future large-scale prospective studies using standardized sepsis definitions, IPF measurement techniques, and validated cut-off values are needed to confirm its clinical utility.

## Author contributions

Conceptualization: SW, IPYP; Data curation: IBAPM, IGPS; Formal analysis: SW, IPYP; Investigation: IBAPM, IGPS; Methodology: IBAPM, IGPS, AAWL; Resources: SW, IPYP; Software: IBAPM, IGPS; Supervision: AAWL; Validation: IBAPM, IGPS, AAWL; Visualization: IBAPM, IGPS, AAWL; Writing–original draft: SW, IPYP; Writing–review & editing: all authors. All authors read and approved the final manuscript.

## Data availability

The data that support the findings of this study are available from the corresponding author upon reasonable request.

## Conflicts of interest and sources of funding

The authors state that there are no conflicts of interest to disclose.

## References

[1] Feng Z., Wang L., Yang J., Li T., Liao X., Kang Y. (2025). Sepsis: the evolution of molecular pathogenesis concepts and clinical management. MedComm.

[2] Rudd K.E., Johnson S.C., Agesa K.M., Shackelford K.A., Tsoi D., Kievlan D.R. (2020). Global, regional, and national sepsis incidence and mortality, 1990-2017: analysis for the Global Burden of Disease Study. Lancet.

[3] Purba A.K.R., Mariana N., Aliska G., Wijaya S.H., Wulandari R.R., Hadi U. (2020). The burden and costs of sepsis and reimbursement of its treatment in a developing country: an observational study on focal infections in Indonesia. Int J Infect Dis IJID Publ Int Soc Infect Dis.

[4] Gauer R.L. (2013). Early recognition and management of sepsis in adults: the first six hours. Am Fam Physic.

[5] Curtiaud A., Iba T., Angles-Cano E., Meziani F., Helms J. (2025). Biomarkers of sepsis-induced coagulopathy: diagnostic insights and potential therapeutic implications. Ann Intensive Care.

[6] Harbarth S., Holeckova K., Froidevaux C., Pittet D., Ricou B., Grau G.E. (2001). Diagnostic value of procalcitonin, interleukin-6, and interleukin-8 in critically ill patients admitted with suspected sepsis. Am J Respir Crit Care Med.

[7] Müller B., Becker K.L., Schächinger H., Rickenbacher P.R., Huber P.R., Zimmerli W. (2000). Calcitonin precursors are reliable markers of sepsis in a medical intensive care unit. Crit Care Med.

[8] Dhainaut J.F. (2005). Re-establishing organ function in severe sepsis: targeting the microcirculation. Crit Care.

[9] Sun G.D., Zhang Y., Mo S.S., Zhao M.Y. (2021). Multiple organ dysfunction syndrome caused by sepsis: risk factor analysis. Int J Gen Med.

[10] Srdić T., Đurašević S., Lakić I., Ružičić A., Vujović P., Jevđović T. (2024). From molecular mechanisms to clinical therapy: understanding sepsis-induced multiple organ dysfunction. Int J Mol Sci.

[11] Dhainaut J.F., Shorr A.F., Macias W.L., Kollef M.J., Levi M., Reinhart K. (2005). Dynamic evolution of coagulopathy in the first day of severe sepsis: relationship with mortality and organ failure. Crit Care Med.

[12] Cepinskas G., Wilson J.X. (2008). Inflammatory response in microvascular endothelium in sepsis: role of oxidants. J Clin Biochem Nutr.

[13] Napitupulu O.D.V.A., Budipratama D. (2024). Pathogenesis and management of sepsis patients with disseminated intravascular coagulation. J Prima Med Sains.

[14] Dolmatova E.V., Wang K., Mandavilli R., Griendling K.K (2021). The effects of sepsis on endothelium and clinical implications. Cardiovasc Res.

[15] Iba T., Helms J., Levy J.H. (2024). Sepsis-induced coagulopathy (SIC) in the management of sepsis. Ann Intens Care.

[16] Tsankof A., Tsakiris D.A., Skoura L., Tsiatsiou P., Ztriva E., Ntaios G. (2025). The role of immature platelet fraction and reticulated platelets in stroke monitoring and outcome prognosis: a systematic review. J Clin Med.

[17] Hamad M.A., Schanze N., Schommer N., Nührenberg T., Duerschmied D. (2021). Reticulated platelets-which functions have been established by In vivo and In vitrovitro data?. Cells.

[18] Goel G., Semwal S., Khare A., Joshi D., Amerneni C.K., Pakhare A. (2021). Immature platelet fraction: its clinical utility in thrombocytopenia patients. J Lab Physic.

[19] De Blasi R.A., Cardelli P., Costante A., Sandri M., Mercieri M., Arcioni R. (2013). Immature platelet fraction in predicting sepsis in critically ill patients. Intens Care Med.

[20] Park S.H., Ha S.O., Cho Y.U., Park C.J., Jang S., Hong S.B. (2016). Immature platelet fraction in septic patients: clinical relevance of immature platelet fraction is limited to the sensitive and accurate discrimination of septic patients from non-septic patients, not to the discrimination of sepsis severity. Ann Lab Med.

[21] Türkmen D., Özsoylu S., Akyıldız B.N. (2022). Comparison of the value of immature retyculocyte and immature platelet in the diagnosıs of sepsis. Pediatr Int.

[22] Wu Q., Ren J., Hu D., Jiang P., Li G., Anjum N. (2015). An elevated percentage of reticulated platelet is associated with increased mortality in septic shock patients. Medicine.

[23] Enz Hubert R.M., Rodrigues M.V., Andreguetto B.D., Santos T.M., de Fátima Pereira Gilberti M., de Castro V. (2015). Association of the immature platelet fraction with sepsis diagnosis and severity. Sci Rep.

[24] Jones N., Tridente A., Dempsey-Hibbert N.C. (2021). Immature platelet indices alongside procalcitonin for sensitive and specific identification of bacteremia in the intensive care unit. Platelets.

[25] Liu Q.H., Song M.Y., Yang B.X., Xia R.X. (2017). Clinical significance of measuring reticulated platelets in infectious diseases. Medicine.

[26] Buoro S., Manenti B., Seghezzi M., Dominoni P., Barbui T., Ghirardi A. (2018). Innovative haematological parameters for early diagnosis of sepsis in adult patients admitted in intensive care unit. J Clin Pathol.

[27] Muronoi T., Koyama K., Nunomiya S., Lefor A.K., Wada M., Koinuma T. (2016). Immature platelet fraction predicts coagulopathy-related platelet consumption and mortality in patients with sepsis. Thromb Res.

[28] McInnes M.D.F., Moher D., Thombs B.D., McGrath T.A., Bossuyt P.M., Clifford T. (2018). Preferred reporting items for a systematic review and meta-analysis of diagnostic test accuracy studies: the PRISMA-DTA Statement. JAMA.

[29] McGuinness L.A., Higgins J.P.T. (2021). Risk-of-bias VISualization (robvis): an R package and shiny web app for visualizing risk-of-bias assessments. Res Synth Methods.

[30] Esposito S., Mucci B., Alfieri E., Tinella A., Principi N. (2025). Advances and challenges in pediatric sepsis diagnosis: integrating early warning scores and biomarkers for improved prognosis. Biomolecules.

[31] Stocker M., Fontana M., El Helou S., Wegscheider K., Berger T.M (2010). Use of procalcitonin-guided decision-making to shorten antibiotic therapy in suspected neonatal early-onset sepsis: prospective randomized intervention trial. Neonatology.

[32] McDade T.W., Borja J.B., Kuzawa C.W., Perez T.L.L., Adair L.S. (2015). C-reactive protein response to influenza vaccination as a model of mild inflammatory stimulation in the Philippines. Vaccine.

[33] Pourcyrous M., Korones S.B., Arheart K.L., Bada H.S. (2007). Primary immunization of premature infants with gestational age <35 weeks: cardiorespiratory complications and C-reactive protein responses associated with administration of single and multiple separate vaccines simultaneously. J Pediatr.

[34] Di Mario A., Garzia M., Leone F., Arcangeli A., Pagano L., Zini G. (2009). Immature platelet fraction (IPF) in hospitalized patients with neutrophilia and suspected bacterial infection. J Infect.

[35] Hubert R.M.E., Rodrigues M.V., Andreguetto B.D., Santos T.M., Pereira Gilberti M.D.F., de Castro V. (2015). Association of the immature platelet fraction with sepsis diagnosis and severity. Sci Rep.

[36] Asghar M.B., Akhtar F., Mahmood A., Rafique N., Rana N.A., Bin K.U. (2023). Diagnostic accuracy of immature platelet fraction (IPF) to differentiate between thrombocytopenia due to peripheral destruction versus bone marrow failure. J Coll Physic Surg Pak.

[37] Cybulska A., Meintker L., Ringwald J., Krause S.W. (2017). Measurements of immature platelets with haematology analysers are of limited value to separate immune thrombocytopenia from bone marrow failure. Br J Haematol.

[38] Li J., Li Y., Ouyang J., Zhang F., Liang C., Ye Z. (2020). Immature platelet fraction related parameters in the differential diagnosis of thrombocytopenia. Platelets.

[39] Ali I., Graham C., Dempsey-Hibbert N.C. (2019). Immature platelet fraction as a useful marker in the etiological determination of thrombocytopenia. Exp Hematol.

[40] Levi M., van der Poll T. (2017). Coagulation and sepsis. Thromb Res.

[41] Williams B., Zou L., Pittet J.F., Chao W. (2024). Sepsis-induced coagulopathy: a comprehensive narrative review of pathophysiology, clinical presentation, diagnosis, and management strategies. Anesth Analg.

[42] Benlachgar N., Doghmi K., Masrar A., Mahtat E.M., Harmouche H., Tazi Mezalek Z. (2020). Immature platelets: a review of the available evidence. Thromb Res.

[43] Arpa M. (2025). Immature platelet fraction as a sensitive biomarker in neonatal sepsis: diagnostic performance preceding thrombocytopenia. Children.

[44] Greene L.A., Chen S., Seery C., Imahiyerobo A.M., Bussel J.B. (2014). Beyond the platelet count: immature platelet fraction and thromboelastometry correlate with bleeding in patients with immune thrombocytopenia. Br J Haematol.

[45] Strauss G., Vollert C., von Stackelberg A., Weimann A., Gaedicke G., Schulze H. (2011). Immature platelet count: a simple parameter for distinguishing thrombocytopenia in pediatric acute lymphocytic leukemia from immune thrombocytopenia. Pediatr Blood Cancer.

[46] McDonnell A., Bride K.L., Lim D., Paessler M., Witmer C.M., Lambert M.P. (2018). Utility of the immature platelet fraction in pediatric immune thrombocytopenia: differentiating from bone marrow failure and predicting bleeding risk. Pediatr Blood Cancer.

